# Aberrant Regulation of RAD51 Promotes Resistance of Neoadjuvant Endocrine Therapy in ER-positive Breast Cancer

**DOI:** 10.1038/s41598-019-49373-w

**Published:** 2019-09-10

**Authors:** Yan Jia, Yueshuai Song, Guolei Dong, Chunfang Hao, Weipeng Zhao, Shufen Li, Zhongsheng Tong

**Affiliations:** 1 Department of Breast Oncology, Tianjin Medical University Cancer Institute and Hospital, National Clinical Research Center for Cancer, and Key Laboratory of Cancer Prevention and Therapy, Huanhuxi Road, Tianjin, 300060 China; 2 Department of Otolaryngology Head-Neck Surgery, Beijing Friendship Hospital, Capital Medical University, Yong’an Road, Beijing, 100032 China

**Keywords:** Cancer therapeutic resistance, Computational biology and bioinformatics, Breast cancer

## Abstract

Breast cancer is one of the most common malignant cancers affecting females. Estrogen receptor (ER)-positive breast cancer is responsive to endocrine therapy. Although current therapies offer favorable prospects for improving survival, the development of resistance remains a severe problem. In this study, we explored the resistance mechanisms of ER-positive breast cancer to neoadjuvant endocrine therapy. Microarray data of GSE87411 contained 109 pairs of samples from Z1031 trial, including untreated samples and post-treated samples with neoadjuvant aromatase inhibitor (AI) therapy. The differentially expressed genes (DEGs) were obtained from two different comparisons: untreated samples *versus* post-treated samples with AIs, and post-treated samples sensitive *versus* resistant to AIs. Multiple bioinformatic methods were applied to evaluate biological function, protein-protein network and potential binding between target protein and aromatase inhibitor. Then, regulation of gene expression, DNA methylation and clinicopathological factors of breast cancer were further analyzed with TCGA data. From GSE87411 dataset, 30 overlapped DEGs were identified. Cell division was found to be the main function of overlapped DEGs by functional enrichment and gene ontology (GO) analysis. RAD51 recombinase (RAD51), a key protein of homologous recombination, was detected to interact with BReast CAncer genes 2 (BRCA2). Moreover, according to the docking simulation, RAD51 might potentially bind to AIs. Overexpressed RAD51 was associated with hypermethylation of BRCA2, resistance to AIs and poor overall survival of patients with ER-positive breast cancer. Furthermore, RAD51 was found to be a better indicator than MKI67 for predicting resistance in neoadjuvant setting. The results indicated that methylation of BRCA2 led to incomplete suppression on RAD51, which caused an increased expression of RAD51, subsequently AI-resistance and poor prognosis in ER-positive breast cancer. RAD51 could be a new candidate used as a predicative marker and therapeutic target in neoadjuvant endocrine treatment.

## Introduction

Breast cancer is the most prevalent cancer affecting females in developed countries. It is the second most common cause of cancer-related death in United States^1^. Approximately 75% of breast cancers belong to the subtype estrogen receptor-positive (ER-positive) constituting the main subtype of the disease. ER-positive breast cancer is generally responsive to endocrine treatment^2,3^. Compared to cytotoxic chemotherapy, neoadjuvant endocrine therapy (NET) is initially only administrated to elderly patients with ER-positive breast cancer, especially the patients who are not suitable for systemic chemotherapy or surgery^4^. In recent years, many clinical studies have tested the efficacy of NET and demonstrated a considerable rate of positive response by patients with ER-positive breast cancer. Because of its low toxicity, reconsideration of NET as a valuable option in neoadjuvant treatment is reasonable, especially as monotherapy for appropriate patients, similar to NCT in combination^3,5–8^. However, it has been proved that not all patients with ER-positive cancer are responsive to endocrine therapy (*de novo* resistance). Moreover, some patients with ER-positive cancer who initially respond would later become refractory to endocrine therapy (acquired resistance)^9^. Thus, several strategies including tyrosine kinase inhibitor, multi-kinase inhibitor, or manipulation of growth factor signaling come out. They may provide hope to patients who are suffering from the resistance to endocrine therapy^9^. Unfortunately, the molecular mechanism of resistance remains unclear.

With the wide application of microarray technique, a large number of data are available for the public databases users. Based on integrated bioinformatic approaches, novel, effective and reliable molecular markers are discovered. To uncover the mechanisms of endocrine resistance, we downloaded GSE87411 microarray dataset from NCBI Gene Expression Omnibus (GEO) database (https://www.ncbi.nlm.nih.gov/geo/). The microarray contained 109 pairs of samples from patients in Z1031 trial, including untreated samples and post-treated samples with neoadjuvant aromatase inhibitor (AI) therapy. Z1031 trial is a randomized neoadjuvant phase II trial in postmenopausal women with clinical stage II/III ER-positive breast cancer. The trial was designed to determine which aromatase inhibitor (Anastrozole, Letrozole or Exemestane) or subset of agents should be recommended for future evaluation against chemotherapy in neoadjuvant setting based on differences in clinical response rates after 16 weeks of treatment^8,10,11^. After 16 weeks of therapy, no significant difference was found among three endocrine agents in terms of Ki67 suppression. Furthermore, the efficacy of chemotherapy was lower than expected in ER-positive cancer exhibiting AI-resistance^8,10,11^. However, it provided us an opportunity to discover mechanisms of endocrine resistance in neoadjuvant setting. In this study, we explored the molecular mechanisms of resistance in order to acquire candidate markers to personalize neoadjuvant strategy for ER-positive breast cancer.

## Material and Methods

### Microarray data

Microarray dataset GSE87411 contained paired information of untreated and post-treated samples from 109 cases of ER-positive patients (clinical trial number: NCT00265759)^7,8^. American College of Surgeons Oncology Group (ACOSOG) Z1031 clinical trial enrolled postmenopausal women with stage II or III ER-positive invasive breast cancer. Eligible patients were treated with Exemestane (25 mg, daily), Letrozole (2.5 mg, daily), or Anastrozole (1 mg, daily) for 16 to 18 weeks before surgery. Level of MKI67 (Ki67) was examined from 2 to 4 weeks after treatment. Additional criteria have been described according to previous report^7,8^.

Gene expression profiles of GSE87411 were downloaded from NCBI Gene Expression Omnibus (GEO) database (https://www.ncbi.nlm.nih.gov/geo/). The platform for GSE87411 was GPL6480, which was agilent-014850 whole human genome microarray 4x44 K G4112F^7,12^. GEO2R software (https://www.ncbi.nlm.nih.gov/geo/geo2r/), SangerBox package (http://sangerbox.com/), and MultiExperiment Viewer (http://mev.tm4.org) were performed to process downloaded data. Then, data was calibrated, standardized, and divided into two pairs of groups. The differentially expressed genes (DEGs) were obtained from two different comparisons: untreated samples *versus* post-treated samples with AIs (T-group) and post-treated samples sensitive *versus* resistant to AIs (R-group). The DEGs were screened out by the criteria of *P* value < 0.05.

### Functional enrichment, GO annotation and the cancer genome atlas (TCGA) analysis

The functional enrichment and GO annotations of overlapped DEGs from T-group and R-group were analyzed by Metascape database (http://metascape.org)^13^. In-depth analysis using Kaplan-Meier plotter (http://kmplot.com), Human Protein Atlas (https://www.proteinatlas.org/), UALCAN database (http://ualcan.path.uab.edu), and UCSC Xena database (https://xenabrowser.net/) were performed on TCGA samples^14–17^. Expression profiles of mRNA were obtained by high throughput sequencing (RNAseq), and genome-wide methylation data was obtained by Illumina infinium 450 K beadchips.

### Network pharmacological prediction

The two-dimensional structures of AIs were generated from PubChem (https://pubchem.ncbi.nlm.nih.gov/)^18^. The high-precision docking simulation was performed by SystemsDock (http://systemsdock.unit.oist.jp/) to access with high quality and reliability of the protein-ligand interactions^19,20^.

### Statistical analysis

The Wilcoxon rank-sum and Fisher exact tests were used to analyze the association between gene expression and clinical characteristics. Pearson correlation was applied to calculate different parameters in terms of correlation. T test was performed for the evaluation of differences between groups. The criteria of *P* value < 0.05 was determined to be significantly different. The statistical analyses were conducted in SPSS Statistics 22.

## Results

### Identification of DEGs associated with AI-resistance

**Figure 1:**
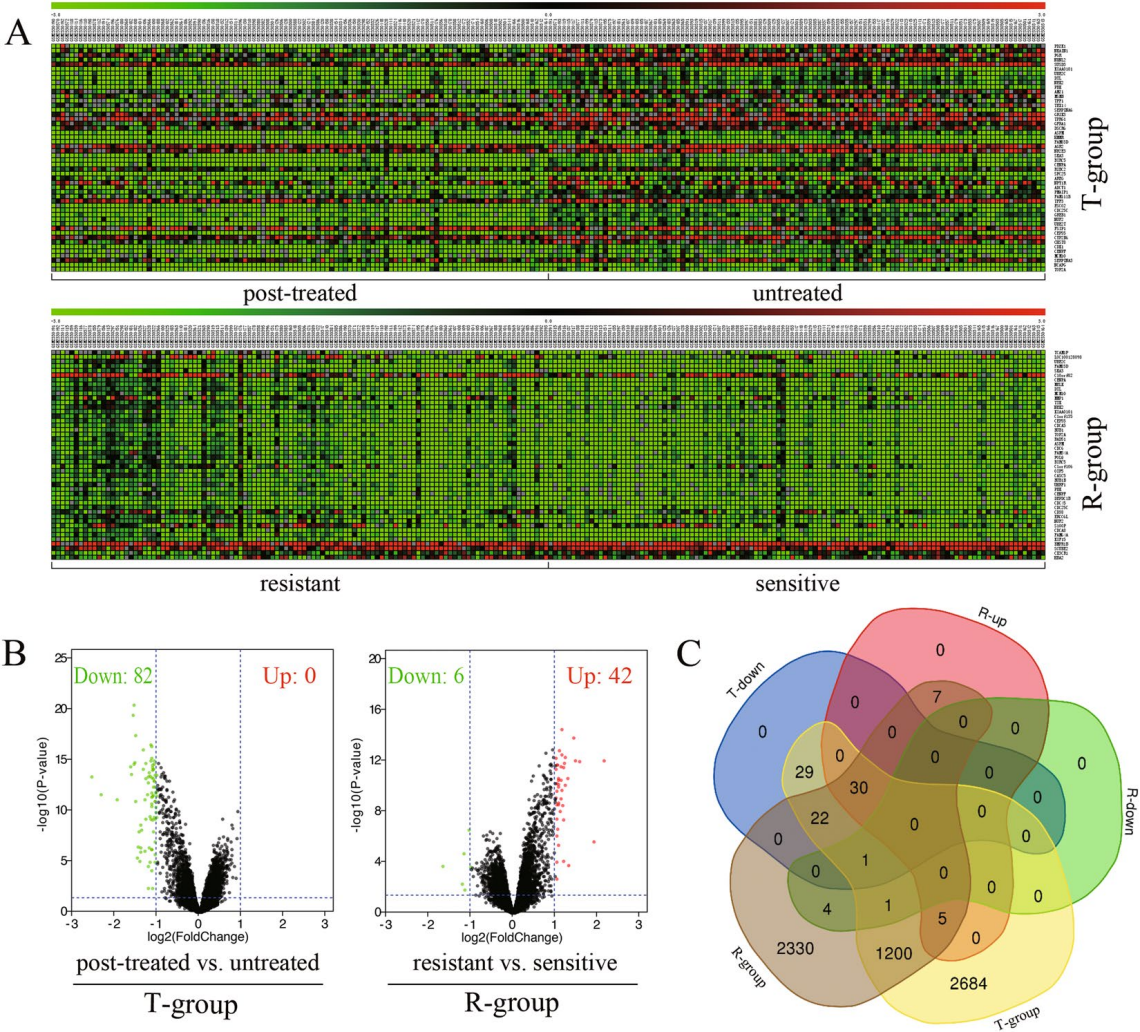
The overlapped DEGs were associated with AI-resistance in ER-positive breast cancer. (**A**) The DEGs of GSE87411 dataset were shown as cluster heatmaps. The DEGs were generated from T-group (untreated samples *versus* post-treated samples with AIs) and R-group (post-treated samples sensitive *versus* resistant to AIs). The red points represented upregulated genes (logFC>1), while the green points represented downregulated genes (logFC<−1). The criteria of *P* value < 0.05 was considered to be significantly different. DEGs, differentially expressed genes; AI, aromatase inhibitor; FC, fold change. (**B**) The volcano plots showed DEGs in T-group and R-group. In T-group, 82 genes were discovered to be downregulated after AI-treatment (T-down), while none was identified to be upregulated. In R-group, 48 DEGs were obtained. Among them, 6 downregulated genes (R-down) and 42 upregulated genes (R-up) were identified. The red points represented upregulated genes, while the green points represented downregulated genes. The DEGs were screened out with the criteria of *P* value < 0.05 and |log fold change (FC)| >1. Down, downregulated genes; Up, upregulated genes. (**C**) The Venn diagram demonstrated different overlaps of genes among 5 groups: T-group, R-group, T-down, R-up and R-down. 30 overlapped DEGs was found to be significantly downregulated after neoadjuvant AI-treatment (T-down), and highly upregulated in AI-resistant samples compared with AI-sensitive samples (R-up). T-down, downregulated genes of T-group; R-up, upregulated genes of R-group; R-down, downregulated genes of R-group.

The GSE87411 microarray data was divided by the GEO2R online software into two pairs of groups, which were defined as T-group (untreated samples *versus* post-treated samples with AIs) and R-group (post-treated samples sensitive *versus* resistant to AIs). The differentially expressed genes (DEGs) obtained from T-group (3972 genes, p < 0.05) and R-group (3600 genes, p < 0.05) were analyzed by SangerBox package and MultiExperiment Viewer. Candidate significantly dysregulated genes were further screened out with the criteria of both *P* value < 0.05 and |log fold change (FC)| >1. Then 82 DEGs were discovered to be dysregulated in T-group. Among them, 82 downregulated genes and 0 upregulated genes were identified. In R-group, we obtained 48 dysregulated DEGs including 6 downregulated genes and 42 upregulated genes. The upregulated and downregulated genes were shown as cluster heatmaps and volcano plots (Fig. 1A,B). Among these dysregulated DEGs, 30 overlapped genes were generated from both T-group (T-down) and R-group (R-up). The expression of overlapped DEGs was significantly downregulated after neoadjuvant AI-treatment, and upregulated in AI-resistant samples compared with AI-sensitive samples (Fig. 1C). The results indicated that the 30 overlapped DEGs were associated with AI-resistance of neoadjuvant therapy.

### Functional enrichment and GO annotations of resistant associated DEGs

**Figure 2:**
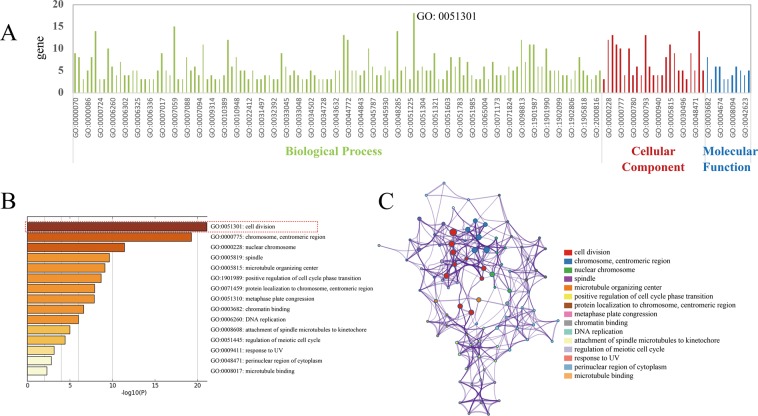
GO annotation and functional enrichment of DEGs revealed 25 genes involved in cell division. (**A**) Based on the analysis of GO annotation and functional enrichment, AI-resistant associated DEGs were divided into three categories including biological processes, cellular component and molecular function. Cell division (GO: 0051301) was the most significant biological process. DEGs, differentially expressed genes; GO, gene ontology. **(B**) GO biological process analysis of AI-resistant associated DEGs was performed by using Metascape with the criteria of *P* value < 0.01. The 25 genes of 30 overlapped DEGs significantly involved in cell division. (**C**) Network plot of the relationships among GO terms. Nodes represented enriched terms colored by its cluster identifier.

Biological annotations and functional enrichment of overlapped DEGs were performed using Metascape database. The results of GO analysis were divided in three categories: biological processes, cellular component and molecular function. Cell division (GO: 0051301) was the most significant biological process (Fig. 2). From the overlapped DEGs, 25 genes (BIRC5, BUB1, BUB1B, CDC25C, CENPA, CENPF, NEK2, TOP2A, UBE2C, OIP5, ERCC6L, CEP55, KNL1, FAM83D, NUF2, CDCA5, SKA3, ASPM, TTK, RAD51, CDC45, MELK, DTL, MCM10 and PBK) were detected in the cell division group (*P* ≦ 10^−10^). It is well known that a dysregulation of these genes can cause malignant diseases by promoting cell division or suppressing normal controls of the cell cycle arrest or the programmed cell death. Thus, uncontrolled cell division and subsequently development of malignant phenotypes are induced under the dysregulation of cell cycle^21^. The results suggested that AI-resistant associated DEGs were involved in cell division and might be related to the regulation of breast carcinogenesis.

### The survival analysis of AI-resistant associated DEGs

To understand the clinical relevance of these 25 genes, the correlation between gene expression and overall survival of patients with breast cancer was further explored. The Kaplan-Meier plotter was utilized to carry out the survival analysis on TCGA breast cancer samples. Among these 25 genes, the expression of CDC25C, ERCC6L or RAD51 was found to be significantly related to overall survival of patients.

**Figure 3:**
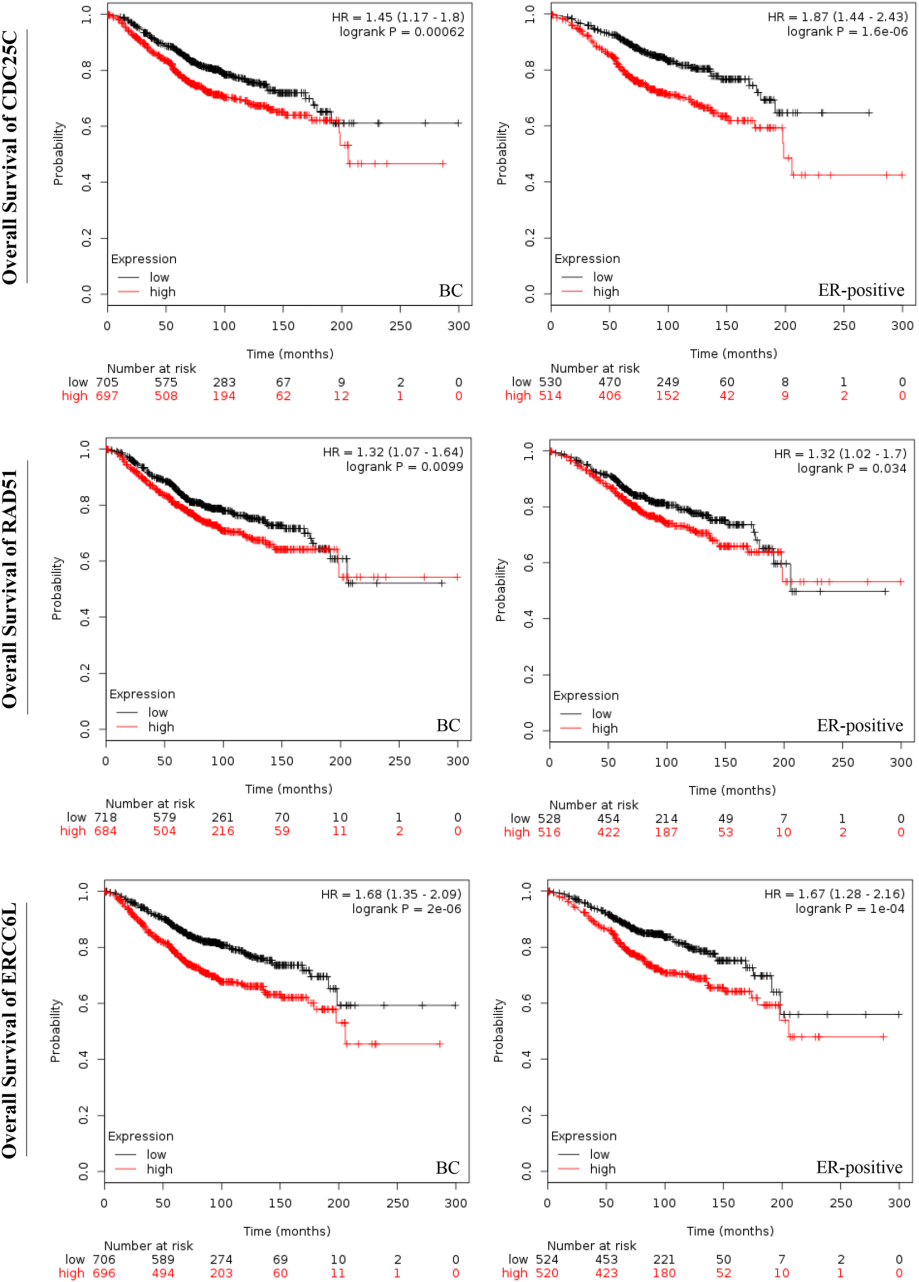
The overall survival of patients with breast cancer or ER-positive breast cancer. The overall survival curve of CDC25C, ERCC6L and RAD51 in breast cancer or ER-positive breast cancer. High expression of RAD51, CDC25C or ERCC6L was related to poor overall survival of patients with breast cancer or specified ER-positive breast cancer during the early follow-up period (before 15 years). The *P* values < 0.05 was considered to be significantly different.

Cell division cycle 25 C (CDC25C) is reported to regulate dephosphorylation of cyclin B-bound CDC2 triggering the entry into mitosis^22^. Excision repair cross-complementation group 6 like (ERCC6L) is a member of the SNF2 family DNA translocase which binds to ultra-fine DNA bridges (UFBs) during mitosis^23^. RAD51 recombinase (RAD51) is known to interact with the ssDNA-binding protein RPA and RAD52 which participate to the homologous recombination repair pathway^24^. As shown in Fig. 3, higher expression of CDC25C, ERCC6L or RAD51 was related to shorter overall survival in breast cancer or specified ER-positive breast cancer (all *P* < 0.05). During the follow-up of patients, the lines corresponding to the high-expression and low-expression were found to be overlapped from 180 to 200 months (almost from 15 to 17 years) in the three plots: CDC25C in BC, RAD51 in BC and RAD51 in ER-positive BC. These results demonstrated that during the early follow-up period (before 15 years), the increased expression of RAD51, ERCC6L and CDC25C could predict an unfavorable overall survival of patients with breast cancer, especially ER-positive breast cancer.

### RAD51 potentially bound to AIs

**Figure 4:**
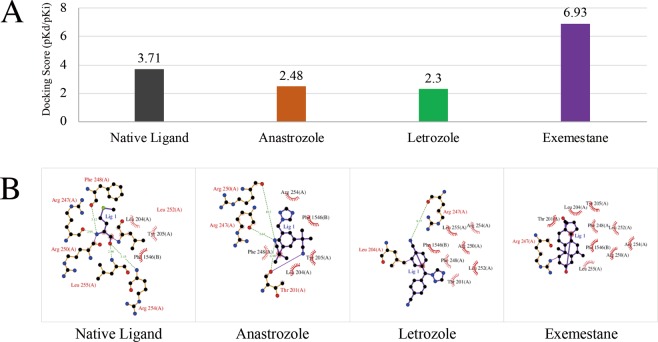
The network pharmacology-based prediction of RAD51 and AIs. (**A**) The docking scores (pKd/pKi) of docking simulation was performed by SystemsDock and shown as bar graph. The docking capacity of Anastrozole, Letrozole or Exemestane for RAD51 was different. (**B**) The 2-dimensional protein-ligand interactions were analyzed by using docking simulation.

To explore the mechanism underlying AI-resistance, SystemsDock, a pharmacology-based prediction network, was used to predict and identify key proteins and their potential interaction with AIs. The docking scores (pKd/pKi) of the docking simulation for RAD51 and AIs were shown in Fig. 4A, while 2-dimensional (2D) protein-ligand interactions of the docking simulation were demonstrated in Fig. 4B. The results showed that only RAD51 possessed the protein structure that was compatible with AIs. Moreover, RAD51 showed a higher docking score with Exemestane compared with Letrozole or Anastrozole. The comparison of the test ligands (AIs) and the native ligand of RAD51 was presented in Table 1. The prediction of SystemsDock demonstrated that most of the residues of the test ligands (AIs) were consistent with the residues of the native ligands. It was well known that aromatase inhibitors markedly suppressed plasma estrogen levels in postmenopausal women by inhibiting or inactivating aromatase^25^. RAD51 was predicted to bind to AIs, which may lead to decreased function of AIs on the inhibition of aromatase. Therefore, the results above suggested that RAD51 had a potential interaction with AIs, especially Exemestane.

**Table 1:** The comparison of test ligands (AIs) and native ligand of RAD51.

Native Ligand	Test Ligands		
	Exemestane (60198)	Anastrozole (2187)	Letrozole (3902)
LEU A 252	LEU A 252	—	LEU A 252
ARG A 250	ARG A 250	ARG A 250	ARG A 250
ARG A 247	ARG A 247	ARG A 247	ARG A 247
PHE A 248	PHE A 248	PHE A 248	PHE A 248
ARG A 254	ARG A 254	ARG A 254	ARG A 254
PHE B1546	PHE B1546	PHE B1546	PHE B1546
LEU A 255	LEU A 255	—	LEU A 255
LEU A 204	LEU A 204	LEU A 204	LEU A 204
TYR A 205	TYR A 205	TYR A 205	—
—	TYR A 201	THR A 201	TYR A 201
9	10	8	9
	(Identical Residues: 9)	(Identical Residues: 7)	(Identical Residues: 8)

### RAD51 was an indicator of poor outcome in breast cancer

**Figure 5:**
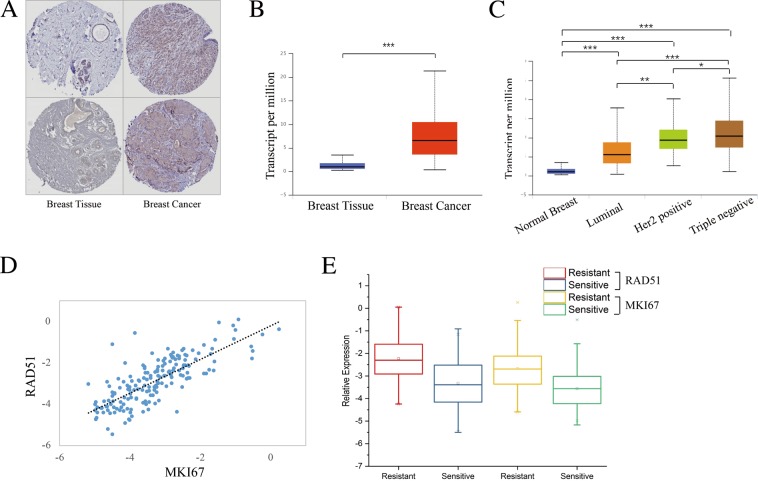
RAD51 was an indicator of poor survival and AI-resistance in breast cancer. (**A**) Representative result of RAD51 expression. Highly expressed RAD51 was found in breast cancer tissues compared to normal breast tissues by immunohistochemistry. (**B**) Highly expressed RAD51 was found in breast cancer tissues (n = 1097) compared to normal breast tissues (n = 114) by analyzing TCGA data. (**C**) An increasing expression of RAD51, in ascending order, was found in luminal (n = 566), Her2-positive (n = 37) and triple-negative breast cancer tissues (n = 116) compared with normal breast tissues (n = 114). (**D**) Positive correlation was found between the expression of RAD51 and MKI67. E. Expression of RAD51 and MKI67 in AI-sensitive samples and AI-resistant samples was shown as box plot. Higher expression of RAD51 and MKI67 was detected in AI-resistant samples than in AI-sensitive samples. In AI-resistant samples, RAD51 had higher expression than MKI67.

In order to explore the roles of RAD51, multiple analyses were performed on TCGA data using UALCAN, UCSC Xena and The Human Protein Atlas database. The result of immunohistochemistry revealed that highly expressed RAD51 was found in breast cancer tissues compared to normal tissues (Fig. 5A). This significant difference was confirmed by the expression analysis of RAD51 in primary breast cancer samples (n = 1097) and normal breast samples (n = 114) (*P* < 10^−12^) (Fig. 5B). For the main subtypes of breast cancer, expression of RAD51 was increasing, in ascending order, in luminal (n = 566), Her2-positive (n = 37) and triple-negative breast cancer tissues (n = 116) compared with normal tissues (n = 114) (all *P* < 0.05) (Fig. 5C). RAD51 was more highly expressed in Her2-positive and triple negative breast cancers (TNBC) than in the other types of breast cancers. TNBC and Her2-positive breast cancers are widely considered as more aggressive cancer types in breast cancer than the luminal one. As we all known, breast cancer is a heterogeneous disease and differs greatly among different patients (intertumor heterogeneity) and even within each individual tumor (intratumor heterogeneity)^26^. As a matter of fact, some luminal cancers also presented unfavorable characteristics, such as resistance to treatment, earlier recurrence and metastasis, and poorer survival. So RAD51 could be a potential predictive marker for ER-positive breast cancer with some unfavorable characteristics.

Moreover, we also found that a high expression of RAD51 was associated with poor survival outcome of patients with breast cancer or ER-positive breast cancer (*P* < *0.01*, *P* < 0.05, Fig. 3). Therefore, all those results suggested that the overexpression of RAD51 was involved in breast carcinogenesis, resistance to AI and had unfavorable impacts on the overall survival of patients affected by breast cancer.

The roles of endocrine therapy mainly rely on the cell-cycle arrest induction instead of the activation of apoptosis. Accordingly, proliferation related markers are commonly used as biological end point to assess the short-term effects of endocrine treatment^27^. In recent years, proliferation marker Ki-67 (MKI67), which was firstly identified by Gerdes in the early 1980s^28^, was considered as a reliable marker in neoadjuvant settings of breast cancer. The rate of cancer cell division was also estimated by performing Ki-67 test. Consequently, in our study, the correlation between the expression of RAD51 and MKI67 was investigated by analyzing GSE87411 dataset. As shown in Fig. 5D, the expression of RAD51 was correlated with expression of MKI67 (Pearson Correlation is 0.773, and *P* < 0.001). Thus, in order to compare the indicative effects on AI-resistance, expression of RAD51 and MKI67 were separately examined in AI-resistant samples and AI-sensitive samples, which were also generated from GSE87411 dataset. A higher expression of RAD51 and MKI67 was observed in AI-resistant samples compared with AI-sensitive samples (Fig. 5E). However, compared to MKI67, RAD51 was found to be a better indicator to predict AI-resistance. Overall, our results suggested that a high expression of RAD51, which was an oncogenic marker, was related to a poor prognostic outcome of breast cancer including shorter survival of patients and AI-resistance in neoadjuvant setting.

### Methylation of BRCA2 decreased its inhibitory effects on RAD51

To figure out the mechanisms underlying RAD51 and AI-resistance, Molecular INTeraction Database was utilized to discover protein-protein interactions between RAD51 and other proteins. It showed that only BRCA2 had direct interaction with RAD51 and *vice versa*. Based on the analysis from Molecular INTeraction Database, the direct protein-protein interaction between RAD51 and BRCA2 was verified by pull down assays. It is reported that BRCA2 directly binds to RAD51 through its BRC repeats and regulates both the intracellular localization and the DNA-binding ability of RAD51. BRCA2 plays multiple roles in the maintenance of genome integrity, especially through homologous recombination (HR)-mediated double-strand break (DSB) repair, which is an important cellular response to DNA damage^29–32^. Therefore, inactivation of BRCA2 leads to a loss of these controls which may be a key event causing genomic instability and tumorigenesis. Thereby, the participation of BRCA2 in RAD51/AI-resistance machinery in ER-positive breast cancer remains to be resolved.

**Figure 6:**
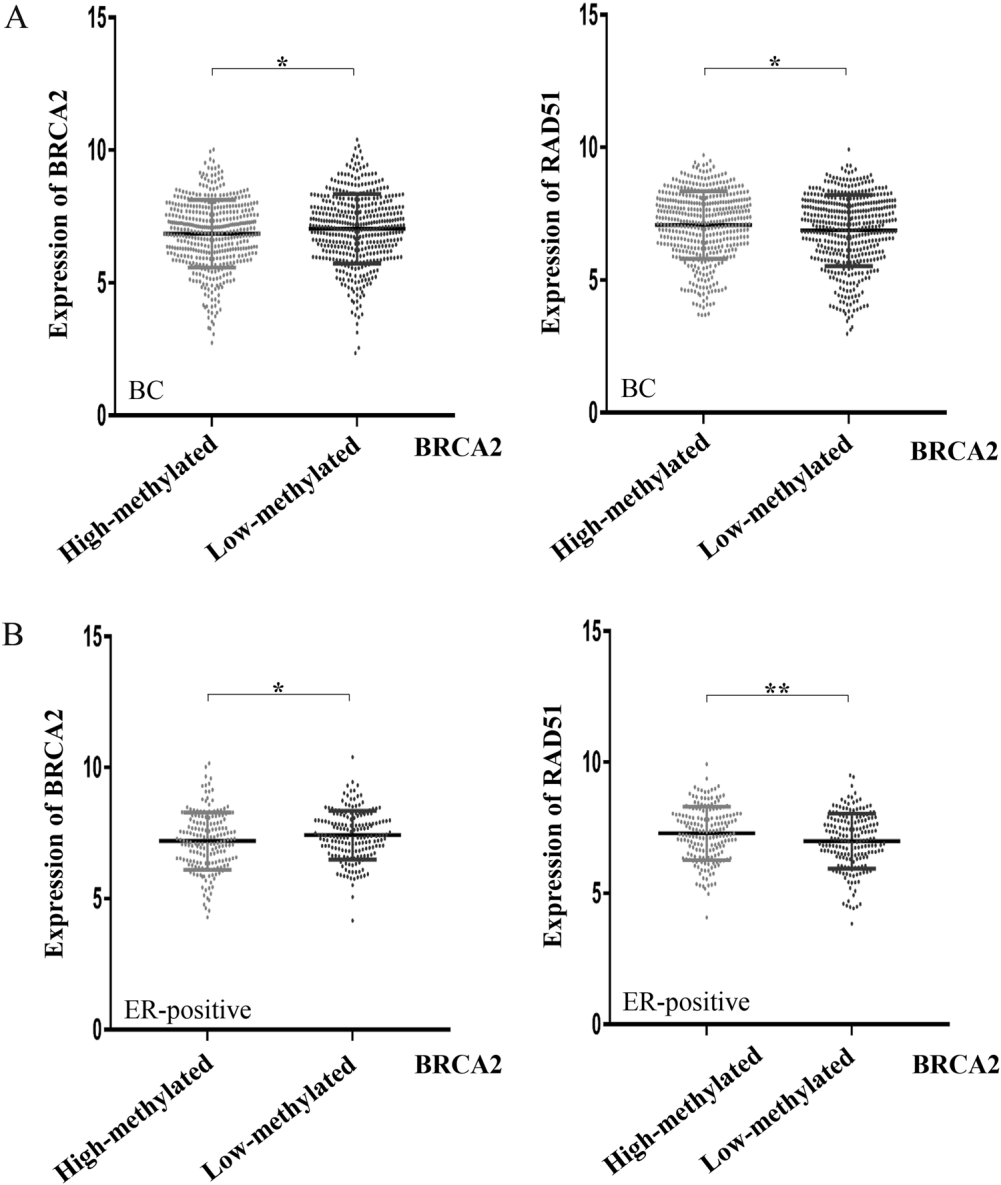
The methylation of BRCA2 was associated with aberrant expression of RAD51. (**A**) Expression of BRCA2 was negatively regulated by the methylation of BRCA2 in breast cancer (*P* < 0.05). Highly expressed RAD51 was detected in breast cancer samples with high-methylated BRCA2 by analyzing the TCGA data (*P* < 0.05). (**B**) In ER-positive breast cancer, decreased expression of BRCA2 and increased expression of RAD51 were found in high-methylated BRCA2 samples by analyzing the TCGA data (*P* < 0.05, *P* < 0.01, separately).

Many studies show that epigenetic modifications including promoter region hypermethylation play important roles in breast cancer^33^. Thus, we explored the relationship between methylation status of BRCA2 and gene expression with TCGA data. To evaluate whether methylation of BRCA2 may decrease its inhibitory effects on the regulation of RAD51, expression of RAD51 and BRCA2 was analyzed in breast cancer samples (n = 873) with low-methylated BRCA2 (n = 436) or high-methylated BRCA2 (n = 437). The results showed that an increased expression of BRCA2 was detected in low-methylated BRCA2 breast cancer samples (Fig. 6A, *P* = 0.037). The expression of BRCA2 was downregulated by the methylation of BRCA2. The epigenetic regulation of BRCA2 had also been proved in previous study^34^. Besides, a decreased expression of RAD51 was found in low-methylated BRCA2 breast cancer samples (Fig. 6A, *P* = 0.022). It suggested that aberrant hypermethylation of BRCA2 was associated with its downregulation of expression but also with an overexpression of RAD51, which may lead to AI-resistance and poor prognosis in breast cancer. Moreover, 363 cases of ER-positive breast cancer (high-methylated BRCA2, n = 182; low-methylated BRCA2, n = 181) were further explored to examine the relationship between the expression of BRCA2 or RAD51 and the methylation status of BRCA2. In ER-positive breast cancer, similar results were obtained as shown in Fig. 6B. The results indicated that an increased expression of BRCA2 and a decreased expression of RAD51 were detected in ER-positive breast cancer samples with low-methylated BRCA2 (*P* = 0.032, *P* = 0.007, separately). All above results demonstrated that the methylation of BRCA2 led to its diminished expression, which caused incomplete suppression of RAD51 thus elevating the expression of RAD51, and hence an unfavorable outcome of breast cancer, especially ER-positive breast cancer.

## Discussion

Breast cancer is one of the main causes of death for female in many countries including USA and Western European countries. About 70–80% of all breast cancers express ERα protein and therefore, endocrine therapy is an important treatment for women suffering from ER-positive breast cancer^35,36^. The main treatment strategies include selective estrogen receptor modulators, aromatase inhibitors and estrogen receptor antagonist. They have been demonstrated to be effective in neoadjuvant, adjuvant, and metastatic settings^37,38^. NET, in postmenopausal women with locally advanced ER-positive breast cancer, may result in a reduction in the tumor size thereby either improving the chances of breast conserving surgery or rendering an inoperable tumor operable^39^. During the last decades, many clinical researches focus on NET of ER-positive breast cancer^8,10,11^. NET is a safe and effective option for localized ER-positive breast cancer. Even as monotherapy, NET has been shown to have a similar response rate to neoadjuvant combination chemotherapy but with significantly lower toxicity. However, not all patients with ER-positive cancer respond to endocrine therapy. Although current treatments offer exciting prospects for better survival, fast development of resistance remains a severe problem. ER-positive breast cancer is a heterogeneous disease, despite its reputation of a relatively benign course, most cancer-related deaths occur within this subset^40^. Unfortunately, mechanisms of resistance in neoadjuvant setting are still relatively unclear. It is urgently needed to investigate the underlying mechanisms of endocrine resistant breast tumors and thereby, distinguish between patients with endocrine sensitive disease and those whose neoadjuvant chemotherapy could not be avoided. Z1031 trial provides us the unique opportunity to discover potential markers facilitating the choice of strategy for postmenopausal women with ER-positive invasive breast cancer and to explore mechanisms of therapeutic resistance.

In this study, we used gene expression profile dataset from Z1031 trial and multiple bioinformatic methods to deeply analyze microarray data. All paired samples were defined as T-group and R-group. The expression of overlapped 30 DEGs was downregulated after neoadjuvant AI-treatment, and significantly upregulated in AI-resistant samples compared to AI-sensitive samples. It suggested that overlapped DEGs were related to neoadjuvant AI-resistance in ER-positive breast cancer. Results of GO annotation and functional enrichments showed that overlapped DEGs were mainly involved in cell division. It is well known that timely and controlled cell division and differentiation are necessary for growth and assembly of functional, well-proportioned tissues. Uncontrolled cell division causes subsequently the development of malignant tumors^21^. Then, 25 genes were detected to be associated with AI-resistance, participating to cell division and having effects on the regulation of breast carcinogenesis. This finding is also consistent with the knowledge that the dysregulation of cell division leads to cancers^41^. Concerning to clinical relevance of these candidate genes, the correlation between gene expression and survival of patients was further explored with TCGA data. Our results indicated that expression of CDC25C, ERCC6L or RAD51 had important effects on overall survival of patients with ER-positive breast cancer. High expression of CDC25C, ERCC6L or RAD51 was significantly associated with the poor survival outcome of patients. Next, network pharmacology-based prediction was used to identify key proteins and predict potential interaction with AIs. Most residues of test ligands (AIs) were consistent with residues of native ligands for RAD51 (Table 1). Moreover, RAD51 showed a higher docking score with Exemestane than with Letrozole or Anastrozole. RAD51 was predicted to interact with AIs, which may lead to a reduction of the aromatase inhibition by AIs and thereby AI-resistance.

RAD51 is a homologue of the bacterial protein RecA, which is required for meiotic and mitotic recombination and for the repair of double-strand DNA breaks^31,42,43^. In order to explore the potential role of RAD51 in breast cancer, multiple analyses about clinical relevance were performed with TCGA samples. High expression of RAD51 was detected in primary breast cancer tissues compared with normal breast tissues. Expression of RAD51 was progressively increasing in luminal, Her2-positive and triple-negative breast cancer tissues comparing with normal breast tissues. To explore the role of RAD51 in the predication of AI-resistance, the correlation between the expression of RAD51 and MKI67 was further investigated. A positive relationship was discovered between the expression of RAD51 and MKI67. Moreover, compared with MKI67, RAD51 was found to be a better indicator to distinguish AI-resistant breast cancer from all AI-treated breast cancer in neoadjuvant settings. Our study provided evidences that high expression of RAD51 predicted adverse outcomes, including AI-resistance and shorter survival, for patient affected by breast cancer. Firstly, overexpression of RAD51 was found in breast cancer compared to normal breast tissue, which facilitated the clinical application by qPCR detection. Secondly, based on microarray data of Z1031 trial, high expression of RAD51 was associated with AI-resistance. Thirdly, RAD51 was found to be structurally compatible with AIs, which may cause dysfunction of AIs and then AI-resistance of breast cancer. Last but not least, poor prognostic value of RAD51 was confirmed via TCGA data. All these results supported RAD51 as a potential prognostic and therapeutic target of breast cancer, which could promote further fine stratification approach for AI-resistant population with ER-positive breast cancer.

Then to figure out the mechanisms underlying RAD51 and AI-resistance, analysis on protein-protein interactions was performed among RAD51 and other proteins. Results indicated that RAD51 directly interacted with BRCA2. Direct protein-protein interaction was confirmed by co-immunoprecipitation assays and previous published literatures. BRCA2 was known to interact with monomeric RAD51 primarily via conserved BRC domains and to coordinate the formation of RAD51 filaments at double-stranded DNA (dsDNA) breaks^44^. The tumor suppressor BRCA2 played a major role in regulation of RAD51-catalyzed homologous recombination^44,45^. Numbers of recent studies showed both genetic and epigenetic mechanisms playing important roles during breast tumorigenesis^46^. Therefore, we analyzed the expression and methylation of BRCA2 in TCGA data. A decreased expression of BRCA2 was detected in high-methylated BRCA2 breast cancer samples and *vice versa*. Results indicated that BRCA2 was downregulated epigenetically. The BRC repeats were demonstrated to be the primary sites through which BRCA2 bound to RAD51^31,42,47^. Moreover, the activity of RAD51 in nucleoprotein filament formation was suppressed by its interaction with peptides encoding BRC repeats^48,49^. Based on above results, the epigenetic regulation of BRCA2 may decrease its inhibitory effects on filament formation of RAD51 and thus impair its ability to recruit RAD51 to dsDNA breaks during homologous recombination. Furthermore, a decreased expression of RAD51 was found in ER-positive breast cancer with low-methylated BRCA2, which was consistent with our hypothesis. Therefore, all the results suggested that aberrant hypermethylation of BRCA2 was associated with its own transcriptional downregulation and the overexpression of RAD51, which finally led to AI-resistance in ER-positive breast cancer. In addition, many genes including BRCA1/2 are involved in the homologous recombination repair (HRR) pathway. A deficiency of those genes and the HRR pathway could lead to synthetic lethality with Olaparib. Olaparib, an inhibitor of poly(ADP) ribose polymerase (PARP), causes PARP to be trapped onto DNA repair intermediates. The inhibition of PARP has an effect on the cells which are deficient in the repair of DNA double-strand breaks by homologous recombination^50,51^. Theoretically, PARP inhibitors may have an effect on breast cancers with deficiency of HRR pathway including a dysregulation of BRCA2/RAD51 machinery.

With the help of integrated bioinformatic analysis, molecular mechanisms underlying resistance in breast cancer will be well understood, and potential targets for predicting prognosis and optimizing treatment will be provided. The results showed that high expression of RAD51 was an adverse indicator for ER-positive breast cancer with neoadjuvant endocrine therapy, including AI-resistance and poor survival outcome. Our data provided supports for additional studies toward the development of enhanced approaches for cancer treatment based on aberrant regulation of RAD51, which may become a potential predicative marker and therapeutic target in neoadjuvant endocrine therapy.

### Ethical standards

All data in this manuscript was collected under the guidelines approved by Tianjin Medical University Cancer Institute and Hospital’s institutional review board and complying with the current laws in China.

## Data Availability

All data is provided in the manuscript.
